# New Instruments

**Published:** 1874-07

**Authors:** 


### NEW INSTRUMENTS.

We have had in use for a few weeks some very fine excavators manufactured by A. Whittington and Co., dental instrument makers, of Louisville, Ky. In form they are very good indeed—having a delicacy and definiteness of shape that is scarcely excelled ; and in temper we have never seen them equalled. We have also some burs for the Morrison engine that are snpe.ior in some respects to anything we have before had in that line ; the shape of the cylinder burs, especially, is worthy of note ; the temper of these is also very good.

We have been using a number of these burs and some very small ones too, and have not yet broken one, which we are not able to say with a similar trial of the burs of any other maker.

One of the greatest annoyances in the use of the Morrison engine has been the breakage of small burs, to such an extent has this occurred in our own hands that we have often almost resolved to abandon their use; but if Whittington's burs are uniformly as good as these the objection will be set aside. We say try them. The trade will be supplied with them.

